# Localization of lipoprotein lipase and GPIHBP1 in mouse pancreas: effects of diet and leptin deficiency

**DOI:** 10.1186/1472-6793-12-14

**Published:** 2012-11-27

**Authors:** Rakel Nyrén, Chuchun L Chang, Per Lindström, Anastasia Barmina, Evelina Vorrsjö, Yusuf Ali, Lisa Juntti-Berggren, André Bensadoun, Stephen G Young, Thomas Olivecrona, Gunilla Olivecrona

**Affiliations:** 1 Department of Medical Biosciences/Physiological Chemistry, Umeå University, Umeå, Sweden; 2 Institute of Human Nutrition, College of Physicians and Surgeons, Columbia University, New York, NY, USA; 3 Department of Integrative Medical Biology (IMB), Umeå University, Umeå, Sweden; 4 The Rolf Luft Research Center for Diabetes and Endocrinology, Karolinska Institutet, Stockholm, Sweden; 5 Division of Nutritional Science, Cornell University, Ithaca, NY, USA; 6 Department of Medicine, David Geffen School of Medicine, University of California, Los Angeles, CA, USA

**Keywords:** Lipoprotein lipase, Diabetes mellitus, Islet cells, Exocrine pancreas, Endothelium, *Ob/ob* mice, High fat diet, Heparin, qPCR, Immunofluorescence

## Abstract

**Background:**

Lipoprotein lipase (LPL) hydrolyzes triglycerides in plasma lipoproteins and enables uptake of lipolysis products for energy production or storage in tissues. Our aim was to study the localization of LPL and its endothelial anchoring protein glycosylphosphatidylinositol-anchored high density lipoprotein-binding protein 1 (GPIHBP1) in mouse pancreas, and effects of diet and leptin deficiency on their expression patterns. For this, immunofluorescence microscopy was used on pancreatic tissue from C57BL/6 mouse embryos (E18), adult mice on normal or high-fat diet, and adult *ob/ob*-mice treated or not with leptin. The distribution of LPL and GPIHBP1 was compared to insulin, glucagon and CD31. Heparin injections were used to discriminate between intracellular and extracellular LPL.

**Results:**

In the exocrine pancreas LPL was found in capillaries, and was mostly co-localized with GPIHBP1. LPL was releasable by heparin, indicating localization on cell surfaces. Within the islets, most of the LPL was associated with beta cells and could not be released by heparin, indicating that the enzyme remained mostly within cells. Staining for LPL was found also in the glucagon-producing alpha cells, both in embryos (E18) and in adult mice. Only small amounts of LPL were found together with GPIHBP1 within the capillaries of islets. Neither a high fat diet nor fasting/re-feeding markedly altered the distribution pattern of LPL or GPIHBP1 in mouse pancreas. Islets from *ob/ob* mice appeared completely deficient of LPL in the beta cells, while LPL-staining was normal in alpha cells and in the exocrine pancreas. Leptin treatment of *ob/ob* mice for 12 days reversed this pattern, so that most of the islets expressed LPL in beta cells.

**Conclusions:**

We conclude that both LPL and GPIHBP1 are present in mouse pancreas, and that LPL expression in beta cells is dependent on leptin.

## Background

Lipoprotein lipase (LPL) is responsible for the hydrolysis of triglycerides in plasma lipoproteins, generating fatty acids and monoglycerides for uptake in tissues and use in metabolic processes [1,2]. LPL is synthesized and secreted by parenchymal cells such as adipocytes and myocytes, but the enzyme acts at the luminal face of endothelial cells in capillaries where it is anchored to the plasma membrane in a heparin-releasable manner. The mechanism by which LPL is transported from the interstitial spaces surrounding parenchymal cells into capillaries involves an endothelial cell protein, glycosylphosphatidylinositol-anchored high density lipoprotein binding protein 1 (GPIHBP1), that binds LPL at the basolateral surface of capillaries and transports it into the capillary lumen [3]. In the absence of GPIHBP1, LPL is mislocalized to the subendothelial spaces, resulting in severe hypertriglyceridemia [4]. Two structural motifs within GPIHBP1 are important for its ability to bind LPL. Mutations within one of the motifs have been identified in patients with severe chylomicronemia [5-8]. Recently it was demonstrated that the transendothelial transport of LPL is bidirectional [9].

LPL is found in large amounts in heart, skeletal muscle, and adipose tissue, but it is also present in kidneys, lungs, fetal liver, lactating mammary gland and macrophages, as well as in scattered cells of the brain [1]. In addition, LPL is found in the islets of Langerhans [10,11]. In INS-1 cells (clonal cells from a rat insulinoma cell line), high glucose levels stimulate the activity of LPL both in total cell extracts and in the heparin-releasable fractions of LPL that is secreted and associated to the cell surfaces [12]. The function of beta cell-derived LPL is unknown, but the stimulation by glucose of LPL activity suggests that it may contribute to beta cell function (or dysfunction) by increasing delivery of lipids to the islets. Acute exposure of islets to fatty acids potentiates glucose-stimulated insulin secretion [13], but chronic exposure causes impaired insulin responses and beta cell death [14]. In other tissues the expression and activity of LPL is regulated by nutritional and hormonal factors [1,2]. LPL activity is usually affected by insulin resistance, diabetes and obesity, although the mechanisms are not fully resolved. The aim here was to study the relative distribution of LPL and its endothelial transport protein GPIHBP1 in mouse pancreas, and to study effects on LPL and GPIHBP1 expression in pancreas by changes in nutritional state (fed compared to fasted), diet composition (normal chow compared to high-fat diet) and by obesity due to leptin deficiency.

## Methods

### Reagents and buffers

Phosphate buffered saline (PBS) was 0.15 M NaCl containing 0.1 M Na2HPO4 and 0.1 M NaH2PO4 (pH 7.5). TBST buffer consisted of 50 mM Tris–HCl, pH 7.4, 0.15 M NaCl, and 0.1% (w/v) Triton X-100. Paraformaldehyde (PFA) (Sigma-Aldrich, P6148) was diluted in 0.1 M PBS pH 7.5 to a final concentration of 4%. Sucrose for fixation of tissues was from BDH (AnalaR, 10274 7E), and the tissue mounting media Tissue Tek 4583 was from Sakura Finetek. The antibodies were diluted in 10% (v/v) heat-inactivated fetal calf serum (FCS) from Invitrogen. TBST was also used for washing of the slides. The sections were mounted in Vectasheild Mounting medium for Fluorescence (Vector Laboratories, CA 94010). Dalteparin sodium (Fragmin®) 2500 IE/KY anti-Xa/ml, a low molecular weight heparin, was from Pharmacia. Glass slides (Super Frost plus) and cover slips were from Menzel-Gläser (J1800AM).

### Animals and procedures

Wild-type (WT) male mice (C57BL/6), six weeks of age, were fed a chow diet (CRM (E) 801730, SDS). C57BL/6 embryos were harvested at eighteen days of gestation (E18), and the pancreas was removed for immunofluorescence. Some mice were treated with Fragmin® 2500 IE (diluted 1:10 in 0.15 M NaCl), administered intraperitoneally (i.p. 1 ml/mouse), and sacrificed 20 minutes later. The animals were on a 12-hours light/dark cycle with free access to water, unless otherwise stated. All animal procedures were approved by the regional ethical committee on studies involving animal experiments, Umeå, Sweden and the corresponding Columbia University’s Institutional Animal Care and Use Committee.

#### Mice on high fat diet, fasted and re-fed

Two groups of three-month-old C57BL/6 male mice, 9 in each group, were included in the experiment. One group was given regular pellets (Research Diets D12450B, 10% kcal% fat and high in carbohydrates) and the other group was given a high fat diet (HFD) for 10 days (Research Diets D12492, 60% kcal% fat). Food was then removed at 4 p.m. and the animals were fasted overnight. In the morning food was given back to five animals in the HFD-group and 5 animals in the control group and they were re-fed for 3 hours before sacrifice. The remaining 4 animals in each group stayed fasted during the corresponding time.

#### Mice fed a diet rich in saturated fatty acids (SAT) for 12 weeks

Four-weeks old male C57BL/6 mice were fed a HFD containing predominantly saturated fat (SAT, 21% fat) or a control, low fat, diet (chow, 4.5% fat) with compositions as detailed previously for 12 weeks [15]. In this experiment the animals were fasted for 2hours before fixation by perfusion of the whole body. Then the pancreases were taken out and treated as described for the other animals. We examined 5 pancreases from the SAT-group and 5 from the chow group.

#### Ob/ob *mice*

*Ob/ob* mice lack leptin [16]. They were taken from a local colony (Umeå *ob/ob*, Histology and Cell Biology, IMB, Umeå University, Sweden). The *ob/ob* genotype was identified by rapid increase in body weight and rise in blood glucose levels compared to the healthy siblings. All mice (female) were fed standard pellets high in carbohydrates (R3, Lactamin, Sweden). The youngest mice were 5.2 and 5.7 weeks old when sacrificed (in total two animals). In addition we examined 6 pancreases each from three and nine months old *ob/ob* mice obtained at two different experimental occasions. *Ob/ob* mice from The Rolf Luft Research Center for Diabetes and Endocrinology, Karolinska Institutet, Stockholm, Sweden (same original strain as the Umeå colony) were used for qPCR on age related LPL expression (1–4, 8 and 11 months old).

#### *Treatment of* ob/ob *mice with leptin*

*Ob/ob* mice used for the leptin experiment were six months of age, and all were females. In one set of experiments, 4 animals were given leptin for 12 days, while 4 were given 0.15 M NaCl. In another set, 6 animals were treated with leptin and 6 used as controls. Leptin or vehicle was injected i.p. twice every day for 12 days. The starting concentration was 0.4 μg leptin/g body weight. After 2 days the concentration was reduced to 0.2 μg leptin/g for 2 days and finally 0.1 μg/g was given for the remaining 7 days. Body weight and food intake were measured on a daily basis. Blood glucose (Ascensia Elite, Bayer) was measured during leptin treatment. The mice had free access to water and food, standard pellets (R3, Lactamin, Sweden).

#### Isolation of islets

*Ob/ob* mice were sacrificed after anesthesia and the pancreas was dissected out. Six *ob/ob* mice were leptin treated and 6 were used as controls. Age and sex-matched (female) C57BL/6 (n=10) was used for comparison. The pancreas was placed in collagenase solution (1.5 mg/ml Collagenase P, Roche Inc.) and shaken for 18 minutes at 37°C. A Krebs - Ringer medium buffer was used with the following composition in mM: 130 NaCl, 4.7 KCl, 1.2 KH2PO4, 1.2 MgSO4 and 2.56 CaCl2 and supplemented with 1 mg/ml BSA and 3 mM D-glucose. The medium was buffered with 20 mM HEPES and NaOH to reach pH 7.4. Islets were picked, counted, placed in buffer RLT from Qiagen and frozen at −80°C.

### Real-time PCR

RNA was extracted from isolated islets and DNAse treated using an RNeasy Micro Kit from Qiagen (Cat. No. 74004). cDNA was prepared from 20 ng total RNA using Moloney Murine Leukemia Virus Reverse Transcriptase, RNase H Minus (M-MLV RT [H–]) (Fermentas) and pd(N)6 Random Hexamer (Fermentas) in total volume of 20 μl. The expression of LPL was quantified by real time PCR as previously described using Maxima probe/ROX qPCR Master Mix (Fermentas) and the ABI Prism 7000 Sequence Detection System (Applied Biosystems, Foster City, CA, USA), using the same primers and probes [17]. Expression levels were normalized to 18S mRNA using the Eukaryotic18S rRNA Endogenous Control Reagent Set supplied from Applied Biosystems. In the experiment with leptin treatment, one pancreas from a leptin treated *ob/ob* mouse gave low islet count for technical reasons and was discarded. Another mouse in the leptin-treated group died, leaving 4 mice for the leptin–treated group and 6 mice for the untreated group.

Analyses of LPL expression on isolated islets from untreated *ob/ob* mice were done using SYBR-Green with beta actin as reference gene. Four mice were used in each age group, except at three months where 3 mice were used.

### Tissue preparation

For sectioning, the organs were dissected free from contaminating tissue in cold PBS and fixed in 4% PFA (paraformaldehyde), at 4°C for 1–2 hours. The tissue pieces were then placed in 30% sucrose in 0.1 M PBS, pH 7.5, at 4°C overnight. The pieces were mounted in Tissue Tek and quickly frozen on dry ice for storage at −80°C or for direct cryosectioning (8-μm sections, put on Super Frost glass).

### Immunofluorescence microscopy

Tissue sections were first blocked with 10% (v/v) FCS in TBST for 1 hour at room temperature. Then they were incubated overnight at 4°C with primary antibodies diluted in the blocking solution. After washing three times with TBST, secondary antibodies diluted in blocking solution were added and incubated for 1 hour at room temperature. Slides were mounted in Vectasheild Mounting Media for Fluorescence (Vector Labs). Visualization of the stained sections was made with a Nikon confocal microscope Eclipse E800, Japan, with the software EZ-C1 Digital Eclipse and Nikon ACT-1. The pictures were taken with magnification 10x, 40x and 60x.

### Antibodies

The anti-LPL antibody was raised by immunizing chickens with bovine LPL; affinity-purified IgY was obtained as described [18]. The batch used was diluted 1:400 and was the same as the one previously thoroughly investigated for specificity [3]. Control IgY (purified from a non-immunized chicken) was diluted to the same protein concentration. AlexaFluor 488– or AlexaFluor 594–labeled goat-anti-chicken antibodies from Molecular Probes (A-11039, A-11042) were diluted 1:1000. The endothelium was identified by staining with rat anti-PECAM (CD31) antibodies from BP Pharmagen (553371) diluted 1:250, and Alexa Fluor 594 goat-anti-rat (A-11007). Islet cells were visualized with antibodies against glucagon (alpha cells), insulin (beta cells), somatostatin (delta cells) and PP-cells. Rabbit anti-glucagon from Linco (403101F) was diluted 1:1000, and visualized with Alexa Fluor 488 or 594 goat-anti-rabbit from Molecular Probes (A-11034, A-11076). Guinea pig-anti-insulin (from Dako, A0564) was diluted 1:500 and visualized with Alexa Fluor 488 or 594 goat-anti-guinea pig antibodies from Molecular Probes (A-11073, A-11076). Rat-anti-somatostatin from Biogenesis (83300009) was diluted 1:500 and visualized with Alexa Fluor 594 goat-anti-rat. Guinea pig-anti-pancreatic polypeptide from Linco (404101) was diluted 1:500 and visualized with Alexa Fluor 594 goat-anti-guinea pig. Nuclei were stained by DAPI from Sigma (D9542). The GPIHBP1 antibody was raised in a rabbit against recombinant mouse GPIHBP1 (amino acids 23–205 in pQE-30 (QIAGEN) expressed in *E. coli*[4]. An immunoglobulin fraction was purified on a 6His-GPIHBP1-Sepharose column, used at 5 μg/ml and visualized with Alexa Fluor goat-anti-rabbit 488 or 594. Monoclonal rat-anti-mouse GPIHBP1, (11A12 concentration 2.67 mg/ml) diluted 1:1500 was visualized with Alexa Fluor 594 goat-anti-rat.

### Statistics

Data was normalized and presented as mean ± SEM (Kruskal-Wallis). Statistical analysis was performed using Graph Pad Prism 5.

## Results

### LPL in endocrine pancreas

**Figure 1:**
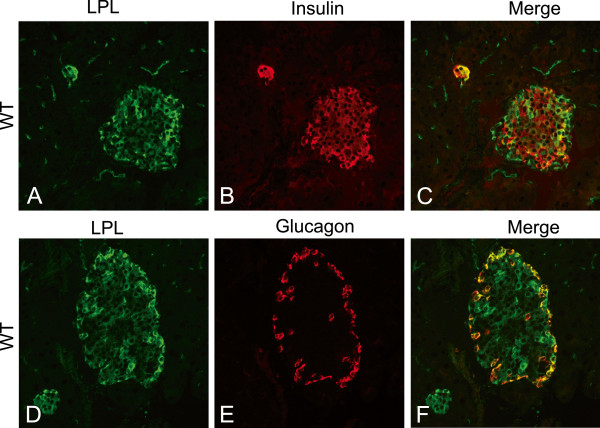
**LPL immune-reactivity in mouse islets.** Islets of Langerhans in pancreas from normal mice (WT) stained with antibodies against LPL and insulin or glucagon. (**A**) Section containing one large and one small islet stained with anti-LPL (green), (**B**) same section stained with anti-insulin to visualize beta cells (red) and (**C**) a merge between A and B demonstrating co-localization between LPL and beta cells. (**D**) Section containing one large and one small islet stained with anti-LPL, (**E**) same section stained with anti-glucagon to visualize alpha cells and (**F**) a merge between D and E demonstrating co-localization between LPL and alpha cells.

Most of the cells in the islets of Langerhans were stained by the anti LPL antibody, but some cells were more fluorescent than others (Figure 1A and D). With antibodies to insulin, co-localization with LPL was evident in most cells in the center of the islets (Figure 1B-C), while cells in the periphery stained only for LPL. With antibodies to glucagon, co-localization with LPL was seen in cells with the peripheral distribution pattern typical for alpha cells (Figure 1D-F). No staining for LPL was seen in the less abundant delta or PP cells (data not shown).

### LPL in embryonic mice at stage E18 and antibody specificity

**Figure 2:**
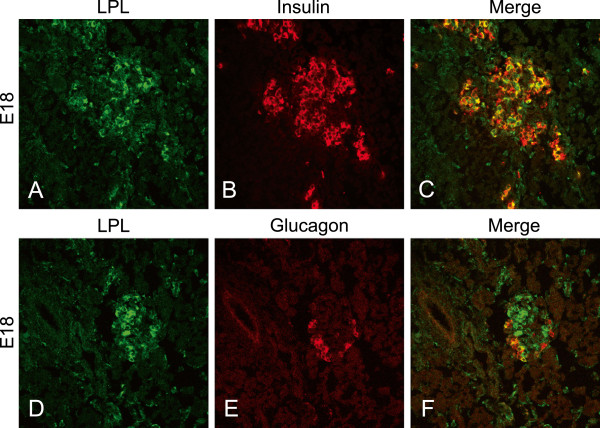
**Immune-reactivity for LPL, insulin and glucagon in islets of embryonic (E18) mice.** (**A**-**C**) Staining for LPL and insulin showing co-localization between LPL and beta cells. (**D**-**F**) Staining for LPL and glucagon showing co-localization between LPL and alpha cells.

It is known that alpha cells tend to show autofluorescence due to their high content of granulae. To investigate whether LPL is really expressed in alpha cells we studied staining for LPL, insulin and glucagon in embryonic pancreases at stage E18 when the alpha cells have not yet developed secretory granules. Immunofluorescence for LPL was seen connected to both beta and alpha cells (Figure 2). The staining for glucagon was weaker than for insulin, but co-staining was visible between glucagon and LPL also at this early stage. This finding supported the results from the adult mice indicating that LPL is expressed both in alpha and beta cells.

### LPL and GPIHBP1 in exocrine pancreas

**Figure 3:**
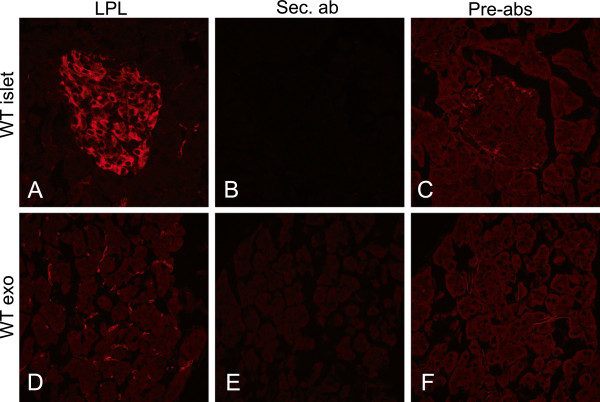
**Controls for specificity of the LPL antibody.** Pancreas from a WT mouse stained with (**A** and **D**) anti-LPL, (**B** and **E**) only secondary antibody and (**C** and **F**) anti-LPL antibody pre-adsorbed with bovine LPL. Exo = exocrine pancreas.

**Figure 4:**
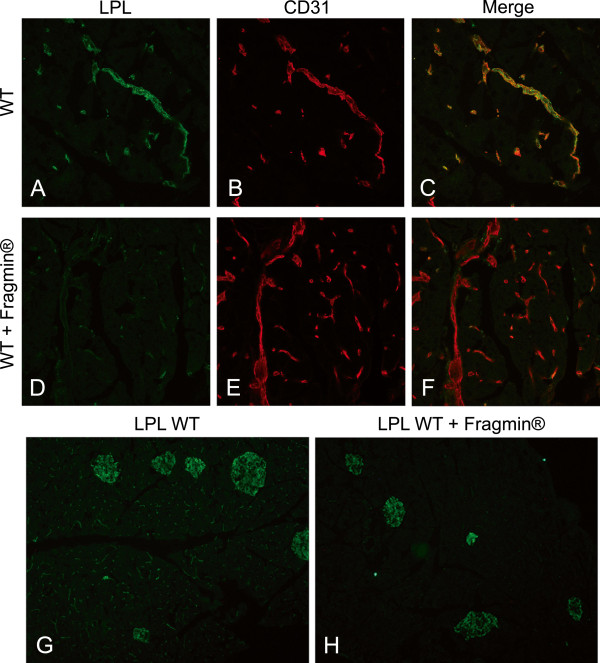
**Release of LPL from blood vessels in exocrine pancreas by heparin injection.** LPL in exocrine pancreas from normal and heparinized (Fragmin®) mice. (**A**) Staining for LPL (green) in a non-treated, WT mouse, (**B**) staining for endothelium of small vessels with anti-CD31 (red) in the same section as A, and (**C**) a merge between A and B. (**D**) Staining for LPL in a Fragmin®-treated mouse. (**E**) Staining with anti-CD31 of the same section as D, (**F**) a merge between D and E. (**G**) Pancreas from a non-treated WT mouse in lower magnification stained for LPL (green) and (**H**) pancreas from a Fragmin®-treated WT mouse stained for LPL.

Next we wanted to exclude the possibility that the staining for LPL was non-specific. Therefore control experiments were made with pre-immune IgY (data not shown), with only secondary antibodies, or with adsorption of the anti-LPL IgY with purified bovine LPL. Compared to the results with anti-LPL IgY, there was little or no fluorescence in either islets or the exocrine pancreas with any of the tested antibodies (Figure 3). In contrast, the positive staining for LPL in exocrine pancreatic tissue with anti-LPL IgY was localized to capillaries, as evidenced by co-localization with the endothelial marker CD31 (Figure 4A-C).

To investigate whether the immune-reactive LPL was heparin-releasable, mice were first injected with low molecular weight heparin (Fragmin®) [19], and were then sacrificed 20 minutes later for studies of LPL in pancreas. Compared to mice that had not been given Fragmin®, much of the LPL immune-reactivity had disappeared from capillaries and small blood vessels of the exocrine pancreas in the Fragmin®-treated mice (Figure 4D), while the staining for CD31 remained associated with the endothelium, as expected (Figure 4E-F). A lower magnification (Figure 4G-H) demonstrated that in the islets the staining for LPL was not much changed in Fragmin®-treated animals compared to non-treated animals. The resistance to Fragmin® indicated that LPL was mostly localized within islets cells. This is in contrast to LPL in exocrine pancreas that was heparin-releasable and therefore presumably exposed on endothelial cell surfaces.

**Figure 5:**
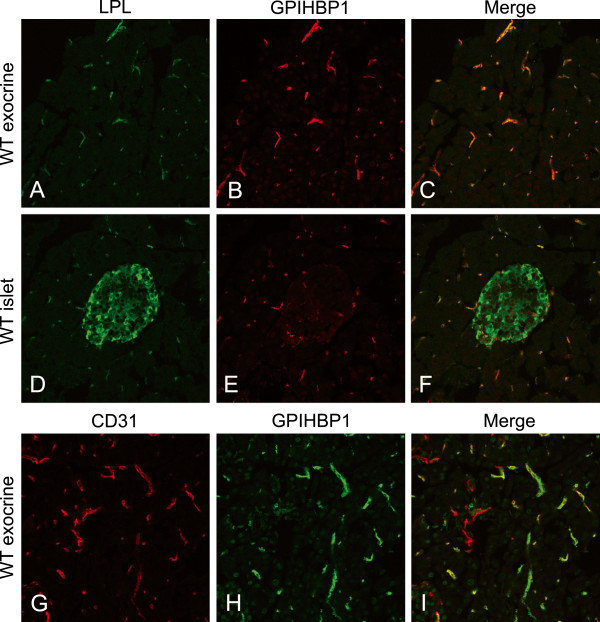
**GPIHBP1 in small blood vessels of exocrine and endocrine mouse pancreas co-localizes with LPL.** (**A**) Exocrine pancreas from a WT mouse stained with anti-LPL (green). (**B**) The same section stained with anti-GPIHBP1 (red) and (**C**) a merge between A and B demonstrating co-localization. (**D**-**F**) Staining for LPL and GPIHBP1 in islet vessels and (**G**-**I**) staining for GPIHBP1 and the endothelial marker CD31 in exocrine pancreas.

Immunostaining for GPIHBP1 (the LPL-binding protein) was associated with capillaries and small vessels in exocrine pancreas (Figure 5B) in a pattern similar to that seen with the anti LPL antibodies (Figure 5A). The staining for GPIHBP1 co-localized with the staining for CD31 (Figure 5G-I). In accord with previous findings in other tissues [4] GPIHBP1 was mostly seen within the smallest blood vessels, but was not present in larger ones. As expected, mice treated with Fragmin® showed the same staining pattern for GPIHBP1 in exocrine pancreas as untreated animals (data not shown). Co-localization between LPL and GPIHBP1 was seen in many of the small blood vessels in the exocrine pancreas and also in the islets vessels (Figure 5A-F).

### LPL in pancreas of *ob/ob* mice and leptin-treated *ob/ob* mice

**Figure 6:**
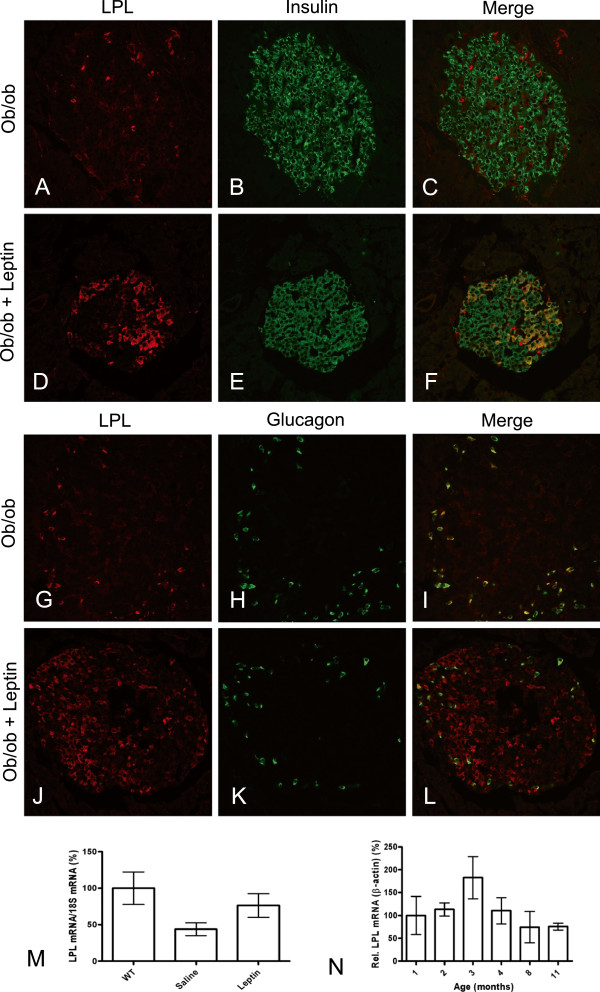
***Ob/ob *mice islets lack LPL in beta cells, but the expression was restored after leptin-treatment.** (**A**) Islet from an *ob/ob* mouse stained with anti-LPL (red), (**B**) with anti-insulin (green), and (**C**) merge of A and B. (**D**) Islet from a leptin-treated *ob/ob* mouse stained with anti-LPL, (**E**) with anti-insulin and (**F**) merge between D and E. (**G**) Another islet from an *ob/ob* mouse stained with anti-LPL, (**H**) with anti-glucagon (green) and (**I**) merge of G and H demonstrating co-localization between LPL and alpha cells. (**J**) Islet from a leptin-treated mouse stained with anti-LPL, (**K**) with anti-glucagon and (**L**) merge between J and K demonstrating LPL reaction in some areas in addition to those containing alpha cells. (**M**) LPL mRNA levels in islets from WT (n=10), saline-treated (n=6) and leptin-treated (n=4) *ob/ob* mice (six months old). Data are presented as mean ± SEM (Kruskal-Wallis p=0.044) and normalized to the results for WT (100%). (**N**) LPL mRNA expression in *ob/ob* mice from one to eleven months of age (n=4 analyzed in each age group except at three months, n=3) calculated as relative expression towards beta actin. Values were normalized to one month old *ob/ob* (100%) and are presented as mean ± SEM (Kruskal-Wallis where the lowest p-value was calculated to 0.057 between three and eleven months).

**Figure 7:**
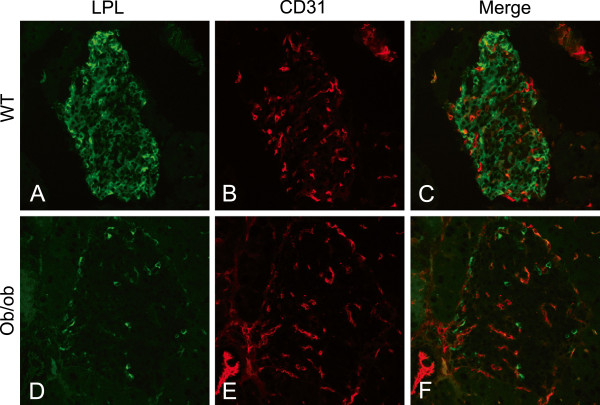
**Islet LPL was mainly intracellular in WT mice but absent in *ob/ob *mice.** Islets from WT and *ob/ob* mice stained for LPL and endothelium (with anti-CD31). (**A**) LPL in an islet from a WT mouse. (**B**) Staining for CD31 and (**C**) a merge showing co-localization between LPL and CD31. (**D**) Pancreatic tissue from an *ob/ob* mouse, LPL staining was only visible in the periphery of the islet (similar to the pattern for alpha cells). (**E**) Anti-CD31 demonstrates small vessels in the islet and (**F**) a merge between D and E showing that most of the LPL reaction was separate from the staining of vessels.

Islets in leptin-deficient *ob/ob* mice are known to be enlarged and to produce increased amounts of insulin [16]. We studied LPL in pancreas of *ob/ob* mice at five weeks and at three, six or nine months of age. In contrast to what was seen in control mice, there were almost no LPL-positive cells in the center of the islets and only scattered LPL-positive cells in the periphery of the islets (Figure 6A and G) at three, six and nine months. Most of the scattered cells stained positively also for glucagon (Figure 6G-I) and hence were presumably alpha cells. The beta cells in *ob/ob* mice stained for insulin, but not for LPL (Figure 6A-C). In five weeks old *ob/ob* mice the islets were smaller than at the older ages, but the pattern for LPL distribution was similar to that in three, six and nine months old animals (data not shown). To study if LPL in the islets was intracellular, or associated with endothelial cells in capillaries, islets from WT and *ob/ob* mice were stained with antibodies to CD31 and LPL (Figure 7). Co-localization was seen between LPL and CD31, but in WT mice the majority of LPL was intracellular in alpha and beta cells. In *ob/ob* mice islets there was very little LPL staining. Some of the immune-reactivity co-localized with extracellular CD31 and the rest with intracellular glucagon.

To investigate whether it was possible to reverse the aberrant pattern for LPL reactivity in islets of *ob/ob* mice towards the pattern seen in WT mice, *ob/ob* mice were treated with daily injections of leptin for 12 days. During this time the treated animals ate less, probably due to the anorectic effect of leptin, and their body weight and plasma glucose levels decreased (Table 1). After treatment with leptin for 12 days, immunofluorescence for LPL was again found in islet beta cells (Figure 6D-F and J). The expression pattern differed from islet to islet. Some islets had recovered almost completely, while others stained for LPL mostly in alpha cells and only in a few beta cells. *Ob/ob* mice in the control group ate more than the leptin-treated group, but still lost some weight, probably due to that they were somewhat disturbed by the daily i.p. injections of saline (Table 1). They did not show any LPL expression in their beta cells. Immune-reaction for LPL was visible in alpha cells in the saline-injected group (Figure 6A and G), just like in *ob/ob* mice at all ages studied. Exocrine tissue of *ob/ob* mice stained for LPL in the same way as that of WT mice, showing co-localization of LPL and CD31 (data not shown). Lean control mice, from the same colony, had a mean weight of 24.8 ± 3.9 g and blood glucose levels of 7.9 ± 0.7 mmol/L (n=4).

**Table 1:** **Weight, food intake and blood glucose levels of *ob/ob *mice before and after treatment with leptin for immunofluorescence**

***Ob/ob *mice leptin or saline**	**Weight before (g)**	**Weight after (g)**	**Total food intake after injections (g)**	**Glucose measure day 2 (mmol/L)**	**Glucose measure day 11 (mmol/L)**
Leptin 1	56.9	46.3	40.8	5.5	4.8
Leptin 2	67.7	59.2	45.9	11.1	3.8
Leptin 3	64.3	52	28.5	7.4	3.7
Leptin 4	62.7	54.5	41.8	5.1	4.5
Control 1	64	56.7	57.9	24.1	31.4
Control 2	58.9	55	74	27.8	23
Control 3	66.9	62.7	84.5	8.8	23.1
Control 4	69.4	68.1	70.5	23.8	25.2

The table demonstrates weight, food intake and glucose levels in ob/ob mice treated with leptin or saline for 12 days. The mice were used for immunofluorescence (IF). All glucose values are from non-fasted animals.

In another comparable experiment mRNA expression analyses were performed on isolated islets. Values from leptin-treated and untreated *ob/ob* mice, compared to WT mice, for blood glucose, weight and food intake are presented in Table 2. LPL mRNA expression was found in both WT and *ob/ob* islets; however, the level of LPL mRNA was significantly lower in islets of saline-injected *ob/ob* mice than in islets from WT mice (p=0.044, Figure 6M). This was consistent with the low or absent immunofluorescence found for LPL in beta cells in *ob/ob* mice. Leptin treatment for 12 days tended to increase the mRNA level for LPL in the islets, but the increase did not reach statistical significance (Figure 6M). There was no significant difference (p>0.05) in the mRNA-levels for LPL in islets from young *ob/ob* mice compared to older *ob/ob* mice (mice were analyzed at 1–4, 8 and 11 months, Figure 6N). The largest difference was seen between three and eleven months with a p-value of 0.057. The mean glucose level in control WT mice was 6.9 ± 0.7 mmol/L (n=10).

**Table 2:** **Weight, food intake and blood glucose levels of *ob/ob *mice before and after treatment with leptin for qPCR**

***Ob/ob *mice leptin or saline**	**Weight before (g)**	**Weight after (g)**	**Total food intake after injections (g)**	**Glucose measure day 0 (mmol/L)**	**Glucose measure day 13 (mmol/L)**
Leptin 1	53.7	43.9	54.2	8.2	6.2
Leptin 2	53.7	43.5	41.3	9.6	4.7
Leptin 3	52.5	45.6	50.8	9.3	5.4
Leptin 4	52	42.5	37.6	7.1	5.6
Control 1	64.6	68.3	89.1	10.2	17.7
Control 2	53.4	55.2	66.9	7.2	6.6
Control 3	49.9	54.1	76.3	10.3	11.1
Control 4	58	60.9	75.1	7.5	10.8
Control 5	52	56	84.1	11.1	14.3
Control 6	48.3	51.5	89.5	6.7	12

The table demonstrates weight, food intake and glucose levels in ob/ob mice treated with leptin or saline for 12 days. The mice were used for determination of mRNA levels by qPCR. All glucose values are from non-fasted animals.

### Effects of high fat diet for 10 days and 12 weeks in WT mice

**Figure 8:**
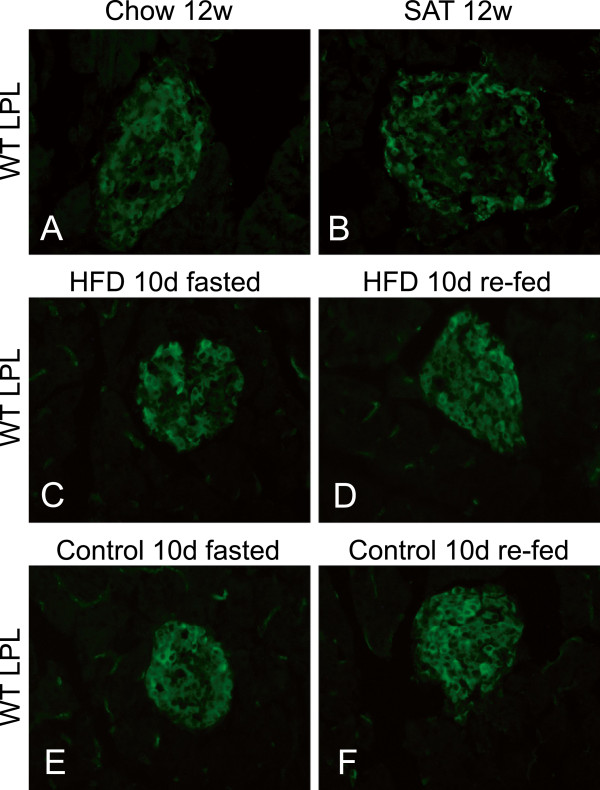
**LPL in islets after high fat diet for 10 days and 12 weeks compared to chow.** (**A**) Pancreas from a mouse fed chow diet stained with anti-LPL. (**B**) Pancreas from a mouse fed SAT diet for 12 weeks. (**C**) Fasted and (**D**) re-fed mice after 10 days of HFD. (**E**-**F**) Fasted and re-fed mice that had remained on chow, for comparison with C and D.

In other tissues than pancreas, both LPL and GPIHBP1 are known to be affected by nutritional and/or hormonal factors [20]. To investigate possible nutritional effects on the distribution of LPL and GPIHBP1 in pancreas we studied WT mice that had been on high fat diet (HFD) for 10 days and compared them to mice on normal chow. Half of the total number of animals in each group (n=10) was sacrificed after overnight fasting, and the other half was re-fed for 3 hours before they were sacrificed. After the different diets there was no difference in blood glucose levels between the ones on HFD for 10 days and the ones on chow diet, but the HFD group weighed 2 g more than the chow group (median, 28 g compared to 26 g). Investigation of their pancreas showed no obvious difference in LPL between the re-fed and the fasted animals or between the ones on HFD for 10 days compared to those on chow diet (Figure 8C-F). Next we investigated pancreases from mice fed a diet high in saturated fatty acids (SAT) for 12 weeks, rendering them hyperlipidemic with regard to plasma triglycerides and non-esterified fatty acids (NEFA) [15], and slightly hyperglycemic (glucose levels were 1.3-fold elevated) compared to mice on normal chow for the same time. A somewhat weaker signal for LPL was seen in the islets from animals on SAT diet compared to animals fed chow diet for 12 weeks, but the differences were not clear enough to be conclusive (Figure 8A-B). The staining for LPL was still mostly in beta cells, both in the SAT-fed mice and in the control mice on chow (data not shown). In both groups the staining for LPL and CD31 in the exocrine parts appeared comparable.

**Figure 9:**
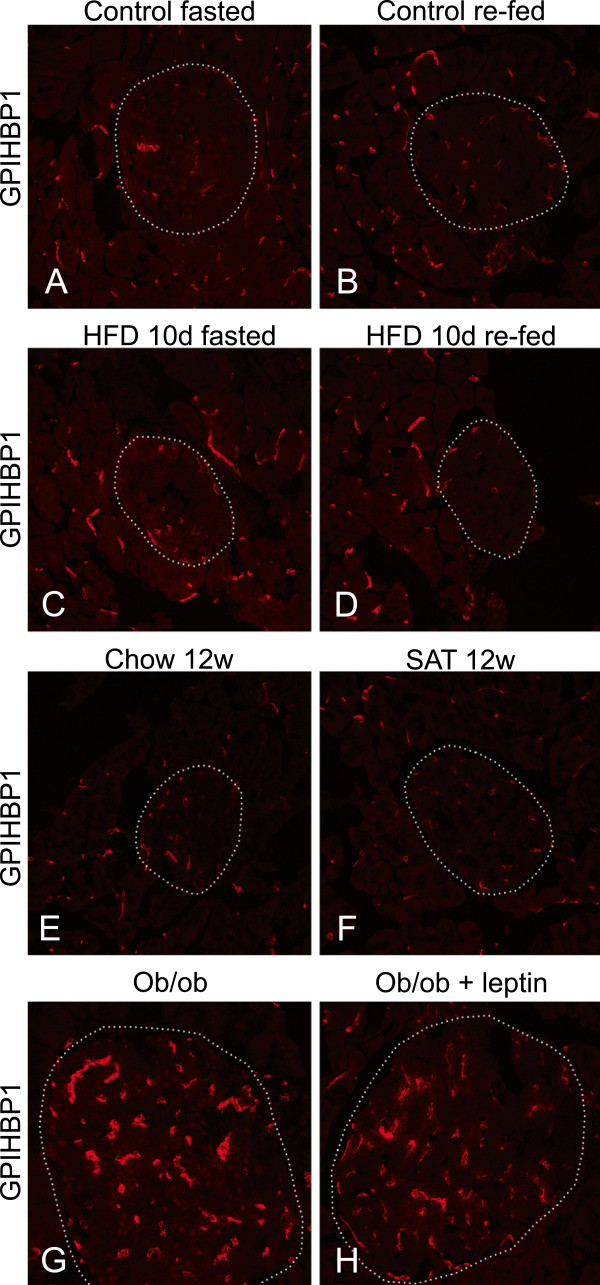
**GPIHBP1 distribution in islets and effects of diet, nutritional status and leptin deficiency.** GPIHBP1 distribution in WT mice who were (**A**) fasted or (**B**) re-fed on normal chow. Mice put on a high fat diet for 10 days and then (**C**) fasted or (**D**) re-fed. (**E**-**F**) Other mice put on a high fat diet for 12 weeks compared to controls on chow. (**G**) GPIHBP1 distribution in *ob/ob* mice compared to (**H**) leptin treated *ob/ob* mice. The dotted lines indicate the islet borders.

**Figure 10:**
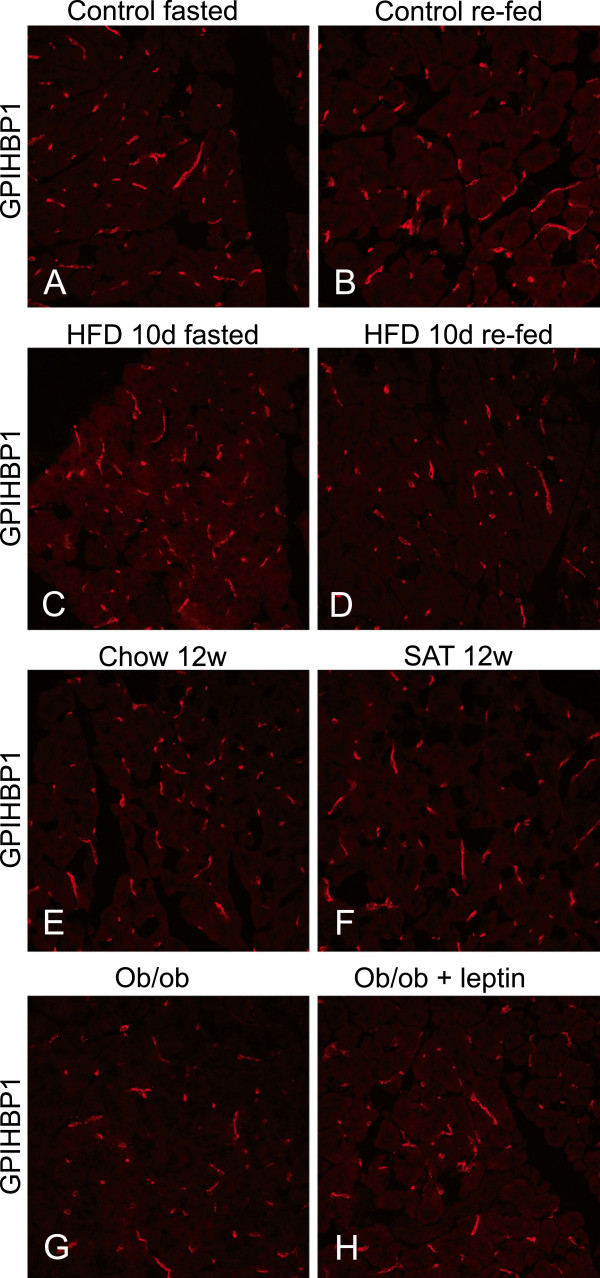
**GPIHBP1 distribution in exocrine pancreas and effects of diet, nutritional status and leptin deficiency.** The sections show exocrine tissue from mouse pancreas and the panels are comparable to those for islets in Figure 9.

The immune-reaction for GPIHBP1 was similar in islets from the fasted and re-fed mice fed a regular diet (Figure 9A-B), and also in those fed a high fat diet for 10 days (Figure 9C-D). The distribution of the immune-reactivity for GPIHBP1 was comparable to the staining for vessels by anti-CD31 (Figure 5). There was no obvious effect on this pattern or intensity after 12 weeks on a diet high in saturated fats (Figure 9E-F). In *ob/ob* mice treated with or without leptin the fluorescence intensity for GPIHBP1 was somewhat increased in the islets (Figure 9G-H). The capillaries and small vessels containing GPIHBP1 appeared wider than in WT mice and this was similar to what was seen with anti-CD31 in islets of *ob/ob* mice (Figure 7E compared to B). In exocrine pancreatic tissue, no effects were seen on the distribution or intensity of GPIHBP1 immune-reactivity by nutritional status, diet or leptin deficiency (Figure 10A-H).

## Discussion

A major new finding in this study is that the expression of LPL in pancreatic beta cells is suppressed in *ob/ob* mice. While there was strong immunostaining for LPL in beta cells from normal mice, there was virtually no staining in beta cells from obese and leptin deficient *ob/ob* mice. This suggests that leptin has an important role for the expression of LPL in beta cells. *Ob/ob* mice eat constantly and become obese, insulin resistant and hyperglycemic. To cope with this they develop islet hyperplasia, and in addition the *ob/ob* mice have a higher proportion of beta cells in their islets than WT mice (>90%) [16]. In contrast to humans with diabetes type 2 due to insufficient insulin secretion [21], the beta cells of *ob/ob* mice have normal glucose-stimulated insulin secretion and produce large amounts of insulin [16]. We found that supplementation of *ob/ob* mice for 12 days with leptin reduced food intake, body weight and blood glucose levels, and restored LPL-expression in the beta cells. Some islets were almost completely restored, while others had only started to recover LPL expression at this time. LPL mRNA levels responded in a similar way. They were lower in *ob/ob* compared to WT mice, and tended to increase in *ob/ob* mice after leptin treatment. The lack of LPL in beta cells of *ob/ob* mice could be due to the lack of leptin *per se*, or could be a consequence of obesity and/or the high levels of glucose, fatty acids and insulin in the blood. A similar response to leptin had been described for hormone sensitive lipase (HSL) in *ob/ob* mice. After 7 days with leptin injections there was increased immunostaining for HSL in islets [22]. Leptin is known to affect LPL in other tissues. In skeletal muscle, but not in adipose tissue, an increase in LPL activity is seen both in WT and *ob/ob* mice after leptin injections [23]. Leptin also affects expression of LPL in J774 macrophages [24]. Taken together, these observations imply an important role for leptin in modulation of the LPL system, and hence in lipoprotein metabolism.

It is known that leptin affects alpha and beta cells through the leptin receptor ObR and that this leads to inhibited insulin and glucagon secretion [25,26]. Pancreas-specific ObR −/− mice show no difference in body weight, food intake or percentage of body fat compared to control mice, while differences are seen in blood insulin concentrations and response to glucose [27]. Like *ob/ob* mice, the pancreas-specific ObR −/− mice develop islet hyperplasia due to increased beta-cell mass. This animal model could be used to investigate whether islet LPL, and HSL, are affected by the local lack of leptin signaling, or by the general metabolic disturbances seen in the *ob/ob* mice.

LPL has previously been shown to be expressed in beta cells and to be closely linked to the function of these cells [11]. Both beta cell-specific overexpression of LPL, and deletion of LPL in beta cells, resulted in a diabetic phenotype [11]. Overexpression of LPL in beta cells decreased islet glucose metabolism, while beta cell-specific knock-out of LPL increased islet glucose metabolism. It is therefore likely that optimal beta cell function requires a delicate balance between metabolism of glucose and fatty acids. In the normal situation, some of the fatty acids may be provided by the LPL system, in addition to the fatty acids that arrive from adipose tissue as albumin-bound NEFAs and fatty acids released from intracellular islet stores by adipose tissue triglyceride lipase (ATGL) and HSL [28]. The intracellular localization of LPL within alpha and beta cells, rather than on the capillary endothelium in the islets, demonstrate that only a minor fraction of the synthesized LPL is secreted from these cells and argues against a function of LPL in lipid uptake by the islet cells. LPL is present in vesicles separate from the insulin-containing granulae in INS1 cells and it was proposed that glucose stimulates LPL translocation to the cell surface [12]. The expression of LPL in INS1 cells has been shown to be up-regulated by agonists to the peroxisome proliferator-activated receptor delta and by cytokines that induce expression of this receptor [29]. We thought that the predominant intracellular localization of LPL in islets could be due to a relative lack of the endothelial LPL-binding protein GPIHBP1. This was not the case. GPIHBP1 was found to be associated with capillaries and smaller vessels both in exocrine pancreas, and in islets. As expected, immune-reaction for GPIHBP1 was co-localized with the endothelial marker CD31 and also with LPL. In islets from *ob/ob* mice the immune-reaction for GPIHBP1 appeared somewhat stronger and the capillaries somewhat wider than in WT mice. After leptin treatment for 12 days, LPL expression was restored in beta cells, but the expression pattern for GPIHBP1 remained the same as in untreated mice, possibly due to that the islets were still enlarged.

An important argument against an involvement of LPL in insulin secretion is that insulin-dependent diabetes is not a commonly reported problem in animal models of LPL deficiency [30,31], or in patients with either total LPL deficiency or with non-functional LPL protein [32]. In one study on human LPL-deficient subjects, enhanced glucose-stimulated insulin secretion after an oral glucose tolerance test was found, compared to other groups of non-diabetic patients with hypertriglyceridemia [33]. Islets from LPL-deficient mice were reported to secrete more insulin than islets from normal mice, and the LPL-deficient mice had lower fasting blood glucose levels and no signs of abnormal insulin responsiveness [10]. With age, LPL-deficient mice were found to display glucose intolerance and decreased first-phase insulin secretion [34]. Thus, a direct effect of LPL on insulin secretion is unlikely, but secondary effects of LPL deficiency on insulin secretion, showing up when the metabolism is in some way challenged, cannot be excluded.

It is well known that the expression of both LPL and GPIHBP1 is regulated by nutritional and hormonal factors. For LPL the adaptation to feeding or fasting occurs mostly by posttranslational mechanism [1,2], while the transcription of GPIHBP1 is upregulated on fasting through activation of peroxisome proliferator-activated receptor-gamma [20]. We studied the response of LPL and GPIHBP1 expression in pancreas of mice exposed to a high fat diet for 10 days or 12 weeks, respectively. The latter group showed increased plasma NEFA and hypertriglyceridemia, indicating insulin resistance [15], while such changes were not yet evident after 10 days on HFD. There were no obvious differences in the LPL distribution pattern in islets of these two groups of diet-treated mice compared to littermates on normal chow. In the 10 day experiment we looked for rapid nutritional effects by comparing LPL distribution in pancreas from fasted mice to those re-fed for 3 hours after fasting. Neither the HFD nor the control groups showed any significant changes in LPL distribution pattern in response to fasting. This was also the case for mice fed a diet containing a high proportion of saturated fat (SAT) for 12 weeks. We conclude that expression and distribution of LPL in mouse pancreatic tissue appears relatively robust with respect to nutritional state and to metabolic disturbances connected to obesity and insulin resistance. A similar conclusion was reached for the expression and distribution of GPIHBP1, but in *ob/ob* mice with or without leptin treatment there is an increase in islet vessel GPIHBP1. This followed the vessel structure in the *ob/ob* islets, which was larger than in islets of WT mice. After leptin treatment for 12 days LPL expression returned in beta cells, but the GPIHBP1 pattern remained the same as in un-treated mice.

In the exocrine pancreatic tissue LPL appeared to co-localize with the endothelial marker CD31 and with GPIHBP1. The function of LPL in exocrine pancreas is not known, but the enzyme is likely to provide the acinar cells with lipolysis products for energy production to support synthesis and secretion of digestive enzymes. In contrast to LPL in islet cells, LPL in exocrine pancreas disappeared after injection of Fragmin®, demonstrating that most of the LPL in exocrine pancreas is localized on cell surfaces in a heparin-releasable manner. This supports the view that the main function of LPL in exocrine pancreas is to act on the triglyceride-rich plasma lipoproteins to release fatty acids and monoglycerides for uptake in the tissue. As expected, GPIHBP1, which is linked to plasma membranes of endothelial cell via a glycosylphosphatidylinositol (GPI) anchor [4], remained at the endothelium after Fragmin® treatment. The source of LPL in pancreatic exocrine tissue is presently not known. We did not see much immunoreaction for LPL over the acinar cells, indicating that they, in contrast to islets cells, secrete most of the newly synthesized LPL. If the acinar cells are not the origin of LPL, endocrine cells might provide exocrine vessels with LPL [35]. In *ob/ob* mice LPL in exocrine pancreas was not affected by the leptin deficiency in beta cells. This argues against the hypothesis that LPL in the exocrine pancreas originates from the islets, unless most of the exocrine LPL comes from alpha cells.

## Conclusions

We have shown that LPL and GPIHBP1 are present on capillaries both in exocrine and endocrine parts of pancreas and that expression of GPIHBP1 is not markedly dependent on either leptin or metabolic status. In islets, LPL is mostly intracellular and LPL expression in beta cells, but not in alpha cells, is dependent on leptin and can be restored after leptin-treatment of *ob/ob* mice. Taken together with earlier studies it is clear that LPL is present within insulin-producing cells, but further studies will be needed to understand its regulation and function.

## Abbreviations

ATGL: Adipose tissue triglyceride lipase; GPIHBP1: Glycosylphosphatidylinositol-anchored HDL-binding protein 1; HSL: hormone sensitive lipase; LPL: Lipoprotein lipase.

## Competing interest

The authors declare that they have no competing interest.

## Authors’ contributions

RN and GO did the conception and design of the research. CLC, PL, AB, YA, LJ-B and TO conducted the animal experiments. RN, EV and YA performed the analytical work and AB and S.G.Y. provided necessary knowledge and tools for studies of GPIHBP1. All authors contributed to analyses of data and to interpretation of the results. RN prepared the figures and RN and GO drafted the manuscript. All authors contributed to edition and revision of the manuscript. All authors read and approved the final manuscript.

## References

[B1] Wang H Eckel RH Lipoprotein lipase: from gene to obesity Am J Physiol Endocrinol Metab 2009 297 E271 E288 10.1152/ajpendo.90920.2008 19318514

[B2] Olivecrona T Olivecrona G Ehnholm C The ins and outs of adipose tissue Cellular lipid metabolism 2009 Heidelberg: Springer 315 369

[B3] Davies BS Beigneux AP Barnes RH 2nd Tu Y Gin P Weinstein MM Nobumori C Nyren R Goldberg I Olivecrona G GPIHBP1 is responsible for the entry of lipoprotein lipase into capillaries Cell Metab 2010 12 42 52 10.1016/j.cmet.2010.04.016 20620994 PMC2913606

[B4] Beigneux AP Davies BS Gin P Weinstein MM Farber E Qiao X Peale F Bunting S Walzem RL Wong JS Glycosylphosphatidylinositol-anchored high-density lipoprotein-binding protein 1 plays a critical role in the lipolytic processing of chylomicrons Cell Metab 2007 5 279 291 10.1016/j.cmet.2007.02.002 17403372 PMC1913910

[B5] Beigneux AP Franssen R Bensadoun A Gin P Melford K Peter J Walzem RL Weinstein MM Davies BS Kuivenhoven JA Chylomicronemia with a mutant GPIHBP1 (Q115P) that cannot bind lipoprotein lipase Arterioscler Thromb Vasc Biol 2009 29 956 962 10.1161/ATVBAHA.109.186577 19304573 PMC2811263

[B6] Olivecrona G Ehrenborg E Semb H Makoveichuk E Lindberg A Hayden MR Gin P Davies BS Weinstein MM Fong LG Mutation of conserved cysteines in the Ly6 domain of GPIHBP1 in familial chylomicronemia J Lipid Res 2010 51 1535 1545 10.1194/jlr.M002717 20026666 PMC3035517

[B7] Franssen R Young SG Peelman F Hertecant J Sierts JA Schimmel AW Bensadoun A Kastelein JJ Fong LG Dallinga-Thie GM Beigneux AP Chylomicronemia with low postheparin lipoprotein lipase levels in the setting of GPIHBP1 defects Circ Cardiovasc Genet 2010 3 169 178 10.1161/CIRCGENETICS.109.908905 20124439 PMC2858258

[B8] Coca-Prieto I Kroupa O Gonzalez-Santos P Magne J Olivecrona G Ehrenborg E Valdivielso P Childhood-onset chylomicronaemia with reduced plasma lipoprotein lipase activity and mass: identification of a novel GPIHBP1 mutation J Intern Med 2011 270 224 228 10.1111/j.1365-2796.2011.02361.x 21314738

[B9] Davies BS Goulbourne CN Barnes RH 2nd Turlo KA Gin P Vaughan S Vaux DJ Bensadoun A Beigneux AP Fong LG Young SG Assessing mechanisms of GPIHBP1 and lipoprotein lipase movement across endothelial cells J Lipid Res 2012 53 2690 2697 10.1194/jlr.M031559 23008484 PMC3494248

[B10] Marshall BA Tordjman K Host HH Ensor NJ Kwon G Marshall CA Coleman T McDaniel ML Semenkovich CF Relative hypoglycemia and hyperinsulinemia in mice with heterozygous lipoprotein lipase (LPL) deficiency. Islet LPL regulates insulin secretion J Biol Chem 1999 274 27426 27432 10.1074/jbc.274.39.27426 10488074

[B11] Pappan KL Pan Z Kwon G Marshall CA Coleman T Goldberg IJ McDaniel ML Semenkovich CF Pancreatic beta-cell lipoprotein lipase independently regulates islet glucose metabolism and normal insulin secretion J Biol Chem 2005 280 9023 9029 15637076 10.1074/jbc.M409706200

[B12] Cruz WS Kwon G Marshall CA McDaniel ML Semenkovich CF Glucose and insulin stimulate heparin-releasable lipoprotein lipase activity in mouse islets and INS-1 cells. A potential link between insulin resistance and beta-cell dysfunction J Biol Chem 2001 276 12162 12168 10.1074/jbc.M010707200 11154699

[B13] Yaney GC Corkey BE Fatty acid metabolism and insulin secretion in pancreatic beta cells Diabetologia 2003 46 1297 1312 10.1007/s00125-003-1207-4 13680127

[B14] Newsholme P Keane D Welters HJ Morgan NG Life and death decisions of the pancreatic beta-cell: the role of fatty acids Clin Sci (Lond) 2007 112 27 42 10.1042/CS20060115 17132138

[B15] Chang CL Seo T Matsuzaki M Worgall TS Deckelbaum RJ n-3 fatty acids reduce arterial LDL-cholesterol delivery and arterial lipoprotein lipase levels and lipase distribution Arterioscler Thromb Vasc Biol 2009 29 555 561 10.1161/ATVBAHA.108.182287 19201689 PMC2743131

[B16] Lindstrom P beta-cell function in obese-hyperglycemic mice [ob/ob Mice] Adv Exp Med Biol 2010 654 463 477 10.1007/978-90-481-3271-3_20 20217510

[B17] Ruge T Sukonina V Myrnas T Lundgren M Eriksson JW Olivecrona G Lipoprotein lipase activity/mass ratio is higher in omental than in subcutaneous adipose tissue Eur J Clin Invest 2006 36 16 21 16403005 10.1111/j.1365-2362.2006.01584.x

[B18] Olivecrona T Bengtsson G Immunochemical properties of lipoprotein lipase. Development of an immunoassay applicable to several mammalian species Biochim Biophys Acta 1983 752 38 45 10.1016/0005-2760(83)90230-8 6849966

[B19] Chevreuil O Hultin M Ostergaard P Olivecrona T Biphasic effects of low-molecular-weight and conventional heparins on chylomicron clearance in rats Arterioscler Thromb 1993 13 1397 1403 10.1161/01.ATV.13.10.1397 8399075

[B20] Davies BS Waki H Beigneux AP Farber E Weinstein MM Wilpitz DC Tai LJ Evans RM Fong LG Tontonoz P Young SG The expression of GPIHBP1, an endothelial cell binding site for lipoprotein lipase and chylomicrons, is induced by peroxisome proliferator-activated receptor-gamma Mol Endocrinol 2008 22 2496 2504 10.1210/me.2008-0146 18787041 PMC2582544

[B21] Weir GC Bonner-Weir S Five stages of evolving beta-cell dysfunction during progression to diabetes Diabetes 2004 53 Suppl 3 S16 S21 15561905 10.2337/diabetes.53.suppl_3.s16

[B22] Khan A Narangoda S Ahren B Holm C Sundler F Efendic S Long-term leptin treatment of ob/ob mice improves glucose-induced insulin secretion Int J Obes Relat Metab Disord 2001 25 816 821 11439295 10.1038/sj.ijo.0801628

[B23] Donahoo WT Stob NR Ammon S Levin N Eckel RH Leptin increases skeletal muscle lipoprotein lipase and postprandial lipid metabolism in mice Metabolism 2011 60 438 443 10.1016/j.metabol.2010.03.016 20494377

[B24] Maingrette F Renier G Leptin increases lipoprotein lipase secretion by macrophages: involvement of oxidative stress and protein kinase C Diabetes 2003 52 2121 2128 10.2337/diabetes.52.8.2121 12882931

[B25] Kieffer TJ Habener JF The adipoinsular axis: effects of leptin on pancreatic beta-cells Am J Physiol Endocrinol Metab 2000 278 E1 E14 10644531 10.1152/ajpendo.2000.278.1.E1

[B26] Tuduri E Marroqui L Soriano S Ropero AB Batista TM Piquer S Lopez-Boado MA Carneiro EM Gomis R Nadal A Quesada I Inhibitory effects of leptin on pancreatic alpha-cell function Diabetes 2009 58 1616 1624 10.2337/db08-1787 19401420 PMC2699864

[B27] Morioka T Asilmaz E Hu J Dishinger JF Kurpad AJ Elias CF Li H Elmquist JK Kennedy RT Kulkarni RN Disruption of leptin receptor expression in the pancreas directly affects beta cell growth and function in mice J Clin Invest 2007 117 2860 2868 10.1172/JCI30910 17909627 PMC1994606

[B28] Mulder H Sorhede-Winzell M Contreras JA Fex M Strom K Ploug T Galbo H Arner P Lundberg C Sundler F Hormone-sensitive lipase null mice exhibit signs of impaired insulin sensitivity whereas insulin secretion is intact J Biol Chem 2003 278 36380 36388 10.1074/jbc.M213032200 12835327

[B29] Kharroubi I Lee CH Hekerman P Darville MI Evans RM Eizirik DL Cnop M BCL-6: a possible missing link for anti-inflammatory PPAR-delta signalling in pancreatic beta cells Diabetologia 2006 49 2350 2358 10.1007/s00125-006-0366-5 16896941

[B30] Liu G Ashbourne Excoffon KJ Wilson JE McManus BM Rogers QR Miao L Kastelein JJ Lewis ME Hayden MR Phenotypic correction of feline lipoprotein lipase deficiency by adenoviral gene transfer Hum Gene Ther 2000 11 21 32 10.1089/10430340050016120 10646636

[B31] Christophersen B Nordstoga K Shen Y Olivecrona T Olivecrona G Lipoprotein lipase deficiency with pancreatitis in mink: biochemical characterization and pathology J Lipid Res 1997 38 837 846 9186902

[B32] Brunzell JD Deeb SS Scriver CR B, Beaudet AL, Sly WS, Valle D, Childs B, Kinzler K, Vogelstein B Familial lipoprotein lipase deficiency, apoC-II deficiency, and hepatic lipase deficiency The Metabolic and molecular bases of inherited disease 2001 New York: McGraw-Hill 2789 2816

[B33] Tamasawa N Matsui J Murakami H Tanabe J Matsuki K Ogawa Y Ikeda Y Takagi A Suda T Glucose-stimulated insulin response in non-diabetic patients with lipoprotein lipase deficiency and hypertriglyceridemia Diabetes Res Clin Pract 2006 72 6 11 10.1016/j.diabres.2005.08.010 16256241

[B34] Ding YL Wang YH Huang W Liu G Ross C Hayden MR Yang JK Glucose intolerance and decreased early insulin response in mice with severe hypertriglyceridemia Exp Biol Med (Maywood) 2010 235 40 46 10.1258/ebm.2009.009100 20404017

[B35] Henderson JR Moss MC A morphometric study of the endocrine and exocrine capillaries of the pancreas Q J Exp Physiol 1985 70 347 356 3898188 10.1113/expphysiol.1985.sp002920

